# The Relationship between Residual Amount of Sr and Morphology of Eutectic Si Phase in A356 Alloy

**DOI:** 10.3390/ma12193222

**Published:** 2019-10-01

**Authors:** Wenda Zhang, Shixuan Ma, Zhenhua Wei, Peikang Bai

**Affiliations:** Department of Materials Science and Engineering, North University of China, No.3 Xueyuan road, Taiyuan 030051, China; zwdno@139.com (W.Z.); msx99edu@163.com (S.M.); weizhnuc@163.com (Z.W.)

**Keywords:** hypoeutectic Al-Si alloys, Sr modification, cooling rate, residual amount of Sr

## Abstract

This paper studied the relationship between the residual amount of Sr and the morphology of the eutectic Si phase in A356 obtained through different modification treatment processes; additionally, the cooling rates of molds were studied. The eutectic Si phase revealed a satisfactory modification effect at residual Sr amounts above 0.01 wt % in A356 alloys cast using an iron mould. Complete modification of the eutectic Si phase could be achieved at a Sr additive amount 0.03 wt % in an A356 melt. The addition of higher amounts of Sr (~0.04–0.06 wt %) did not improve the modification effect. With the addition of 0.06 wt % Sr into A356 alloy melt and holding at 750 °C, the anti-fading capacity of Sr modification effect could be sustained for 120 minutes. More Sr is needed to obtain a good modification of eutectic Si for an A356 alloy cast using a sand mold.

## 1. Introduction

Exhibiting excellent mechanical properties at both room and high temperatures, as well as high corrosion and wear resistance, among other advantages, cast Al-Si alloys are extensively used in auto, aerospace, weaponry, and other industries [1,2]. The unmodified eutectic Si phase presented in Al-Si alloys usually exists in a plate-like form incorporated into an aluminum matrix. For this reason, the treatment of cast Al-Si alloy melts usually requires modification treatments to improve the mechanical properties (especially plasticity) [3,4,5]. Recently, Sr-based modification of eutectic Si has basically replaced the traditional approach of Na-based modification, which is widely used nowadays as a modifier for cast Al-Si alloys, because the advantages of Sr-based modification of eutectic Si are long life, non-corrosion lining, low levels of pollution, and good remelting properties.

While the presence and amount of Sr have been verified, it has a great influence on many properties of cast Al-Si alloys, such as the mechanical properties [6], the corrosion behavior [7], and the hot tear sensitivity [8]. However, lots of research and practice shows that during the modification treatment of cast Al-Si alloys of the same grade, different additive amounts of Sr (ranging from 0.01% to 0.06%) frequently give rise to the same modification effect. For instance, Gang et al. [9] reported that adding 200–600 ppm of Sr to an Al-7Si-0.5Mg alloy significantly improved its plasticity. Sigworth [4] argued that in order to achieve the desired modification level, Sr addition should be in the 80–120 ppm range. Furthermore, Sr modification also has obviously different incubation periods. Some studies show that Sr has an incubation period of 1–2 h, while others indicate that this incubation takes several minutes. In fact, processes like melt constitution, melting process (which includes melting temperature, hydrogen content, and refining), etc., all affect the modification to some extent. Additionally, due to the different types and contents of trace elements in alloys, different amounts of Sr have to be added. The alloying degree of cast Al-Si alloys increases with the increasing demand for high-performance, which leads to a very diversified range of alloying [10], as well as impurities in the recycled cast Al-Si alloys. The impurities might poison and/or weaken the modifying effect of the added Sr [11,12]. Thus, to neutralize or offset the harmful effects of some trace elements, it is necessary to add larger amounts of Sr. However, an excessive amount of Sr forms a detrimental Al_2_Si_2_Sr phase [13,14,15], and frequently gives rise to increased hydrogen content in the melt [16,17,18]. Excess Sr can also initiate structural looseness and performance deterioration. Therefore, to obtain high-performance cast Al-Si alloys, optimization of the amount of Sr added as a modifier, taking into account the composition and properties of the raw materials, is needed.

This paper mainly studied the relationship between the residual amount of Sr and the morphology of the eutectic Si phase in a hypoeutectic Al-Si alloy obtained using different amounts of added Sr, as well as different melt holding times and cooling conditions. The result could offer guidance for industrial melting and the casting of Al-Si alloys, especially of Al-Si alloys obtained by melting recycled aluminum scraps.

## 2. Materials and Methods

**Figure 1:**
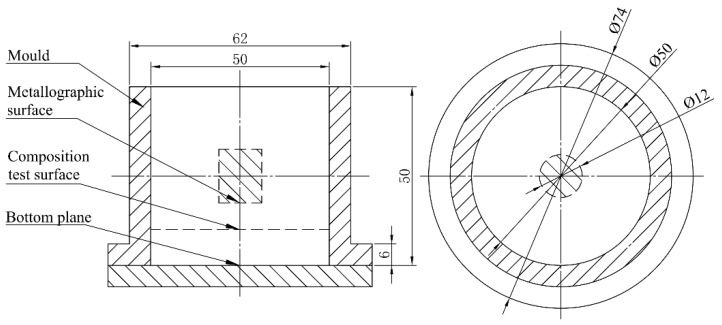
Schematic diagram of taking samples for composition test and microstructural observation (marked with the slash), all the dimension unit in Figure 1 are mm.

Table 1 shows the composition of the A356 (Al-7%Si-Mg) aluminum ingot used for experiments. A SG-5–10 resistance furnace and clay-graphite crucibles were utilized for melting. When the melting temperature reached 750 °C, Sr was added to the 0.5 Kg melt in amounts of 0.00%, 0.02%, 0.03%, 0.04%, 0.05%, and 0.06% (uniformly mass fraction) and in the form of the Al-10Sr master alloy for modification treatment, respectively. Ten minutes after the addition, the melting temperature dropped to 700 ± 5 °C. Alloys were cast into an iron mold (Φ 50 × 50 mm in size) at room temperature. Sand and iron molds commonly used in production were adopted to represent different cooling rates in order to allow us to compare the influence of different cooling rates on the Sr modification effect. To compare the effect of different cooling rates on Sr modification, 0.5 Kg A356 melt with 0.06% of Sr was cast into a cylindrical iron mold and a sand mold, respectively, which are commonly used in production to achieve different cooling rates. To determine the duration of the Sr modification effect, a total of 1.5 kg aluminum melt was prepared. At 750 °C, 0.06% of Sr was added to batches of the alloy, holding for 30, 60, 90, 120, 150, 180, and 240 min; after that, the samples were taken for direct casting into the cylindrical metal mold (at room temperature) which was Φ 50 mm × 50 mm in size. In order to avoid the influence of defects such as pores and the shrinkage cavities formed during the casting process, the composition test surface was taken from the bottom of the cast sample, and the observation surface of samples for metallographic analysis was taken from the lower end of the center of the composition test sample (see Figure 1). 

In general, the thermal analysis method is a non-destructive, convenient, and effective method to judge the modification degree of an Al-Si alloy. Cooling curves and their corresponding first derivative curves were used to assess the solidification behavior and to determine any characteristic temperatures. In this paper, the data for thermal analysis was captured using an 8-channel data acquisition system linked to a personal computer equipped with proprietary software for automated data collection, processing, and analysis. The sampling time interval of the instrument was 0.1 s. In order to avoid the influence of the pouring temperature and cooling rate on the solidification cooling curves of the A356 alloy, the pouring temperature and die temperature remained constant.

Metallographic samples were first ground and polished and then etched with Keller’s reagent (2 ml HF, 3 ml HCl, 5 ml HNO_3_, and 190 ml H_2_O). A Zeiss-Imager metallographic (Zeiss, Oberkochen, Germany) was used for metallographic observation. To reveal the 3D morphology of the eutectic Si particles, the alloys were deeply corroded in a 15% NaOH solution for 20 min at 60 °C. SUPRA55-a field emission scanning electron microscope (FE-SEM) (Zeiss, Oberkochen, Germany) coupled with an Oxford INCA energy-dispersive analyzer (EDS) (Oxford Instruments, Oxford, the United Kingdom) was used to analyze the 3D morphology of the Si particles and the intermetallic phases composition. The chemical analysis of each sample was performed using a SPECTRO direct-reading spectrometer (SPECTRO, Kleve, Germany). 

## 3. Results and Discussion

### 3.1. Influence of the Sr Additive Amount on Modification Effect

**Figure 2:**
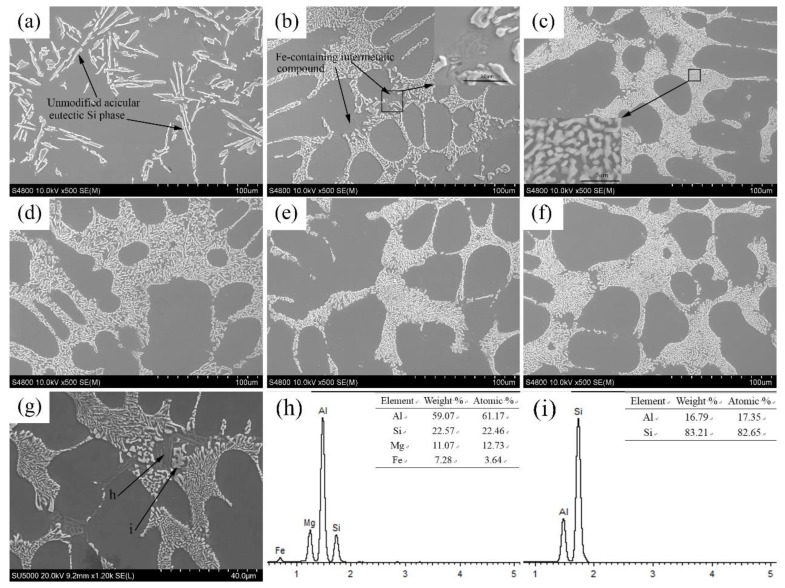
Microstructure of the samples with different Sr additions: (**a**) 0.00%, (**b**) 0.02%, (**c**) 0.03%, (**d**) 0.04%, (**e**) 0.05%, (**f**) 0.06%. (**g**) morphology of iron-rich phase and partially modified eutectic Si, (**h**–**i**) corresponding EDS results.

Figure 2 shows the changes in the morphology of the eutectic Si phase in the A356 aluminum alloy with different Sr additions.

The unmodified eutectic Si phase in the A356 aluminum alloy showed a typical, bulky, acicular morphology (see Figure 2a). When 0.02% of Sr was added, the eutectic Si phase underwent the desired modification (see Figure 2b), as its morphology changed from bulky acicular to fibrous. As the Sr amount increased to 0.03%, the fibrous eutectic Si phase became more refined, and the eutectic field became more compact (see Figure 2c). However, as shown in Figure 2d–f, increasing the amount of Sr (0.04–0.06%) in the aluminum alloys cast from the metal mold did not produce any further improvements, which agrees with the results observed by Zamani et al. [13]. The results of the direct-reading spectrometer indicated that, in constitutional samples cast with an aluminum melt with initial Sr quantities of 0.02%, 0.03%, 0.04%, 0.05%, and 0.06%, the residual amounts of Sr were 0.011%, 0.019%, 0.029%, 0.039%, and 0.044%, respectively. These results suggest that the eutectic Si phase could achieve the desired modification effect at Sr residual amounts above 0.011%.

**Figure 3:**
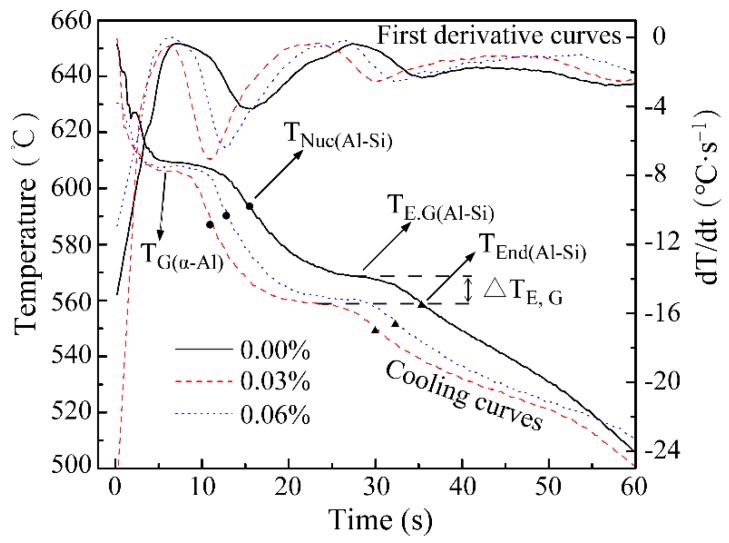
Cooling curves and their first derivative curves of A356 alloy unmodified and modified with 0.03wt.% and 0.06wt.% Sr, respectively.

Figure 3 shows the cooling curves and their first derivative curves of the unmodified and modified with 0.03 wt %, 0.06 wt % Sr A356 alloy, respectively. Five general features can be extracted from the cooling curves assistant by their first derivative curves, namely: The T_G(α-Al)_-Growth temperature of the primary α-phases, T_NUC(Al-Si)_-Nucleation temperature of the eutectic structure, T_E,G_-Eutectic growth temperature, T_End(Al-Si)_-Eutectic end temperature, and ΔT_E,G_ = T_E,G(unmodified)_ − T_E,G(modified)_. Among these parameters, T_NUC(Al-Si)_ and T_E,G_ are greatly affected by the cooling rate, while ΔT_E,G_, the most reliable indicator of the modification level, has been the object of more and more attention [13,19]. The addition of Sr leads to a depression the eutectic growth temperature which is reflected in the increased value of ΔT_E,G_. When the Sr content reached 0.06%, ΔT_E,G_ did not increase further, or even decreased slightly. This is consistent with the analysis of the microstructure, which mean that a further increase in Sr content does not achieve a better modification effect. Excess Sr will form a AlSiSr intermetallic compound which reduces the effect of the modification of Silicon and increases the eutectic temperature of the alloy [13]. Whether in the absence of Sr or not, the recalescence undercooling is hardly distinguished, which is inconsistent with the conclusion of Zamani [13], and therefore may be related to an inconsistency in the alloy’s composition.

As mentioned, some alloying or impurity elements have poison effects on Sr. It was found that Fe has an effect on the modification effect of Sr. Typically, Fe is an unavoidable impurity for Al-Si alloys, as it affects their solidification. Shankar et al. [20] reported that in unmodified hypoeutectic Al-Si alloys, the eutectic Si phase nucleates in the β-Fe phase prior to the nucleation of the eutectic Al phase, in which the Si phase typically grows freely, forming its typical acicular morphology. However, the growth of β-Fe phase is inhibited in chemically-modified hypoeutectic Al-Si alloys; thus, numerous equiaxed eutectic aluminum grains nucleate before the formation of eutectic Si occurs. As a result, eutectic Si is forced to take on a fibrous morphology in the eutectic aluminum phase. 

In Figure 2b–f, incompletely-modified, massive eutectic Si with irregular shapes could be observed uniformly at eutectic Si mass boundaries (as shown by the black arrows in Figure 2b). The EDS of the acicular phases (h) around the partially-modified eutectic Si phases (i) (see Figure 2h–i) demonstrated that these phases contained Fe. The element ratios in these phases were close to those of the Al8FeMg3Si6 phase. Thus, the eutectic Si phases near the Fe-containing phases always exhibited incomplete modification. Lan et al. [21] made similar observations. They concluded that irregular, β-Fe-rich phases provided nucleation sites for eutectic Si phases, causing them to form irregular shapes which are characteristic of an unmodified state. Apparently, it can be inferred that these partially-modified eutectic Si phases are somewhat related to the Fe distribution in the alloy, which, in turn, depends on interactions between the Sr and Fe-rich phases [13,22]. According to Lan et al. [21], the presence of a small amount of Fe in Al-Si alloys weakens the Sr modification capacity, because Fe-rich phases consume some Sr during the solidification process. However, in our research, EDS did not detect Sr in the Fe-rich phases. Furthermore, an incomplete modification was also observed in sites without Fe at the edge of the eutectic structure. Thus, it is unlikely that the unmodified state of eutectic Si at the edge of the eutectic structure was only due to the presence of Fe. Due to some limitations in the experimental techniques, both the metallographic and SEM analyses demonstrated only a 2D section or a local side; thus, it is possible that Fe phases exist in the eutectic region that are hard to detect by these methods. Therefore, further in-depth studies are needed to figure out the effect of Fe-rich phases on Sr-modified eutectic Si phases.

The influence of Sr on the solid-liquid interface also gives rise to changes in the growth state of eutectic Si. On the one hand, the presence of Sr changes the rheology of primary α-Al particles, as well as of the solidification-front eutectic liquid region, and thus, influences the interfacial tension between them, altering the growth mode of the eutectic Si phase. On the other hand, constitutional supercooling of the solid-liquid interface, induced by Sr, changes the morphology of the eutectic Si phase. The degree of constitutional supercooling is usually expressed by a supercooling degree ∆T [23]:

(1) $$∆T=\frac{m_{L}C_{0}\left(1-k_{0}\right)}{k_{0}}\left\{1-exp\left(-\frac{\upsilon }{D_{L}}x\right)\right\}-G_{L}x^{\prime }$$

where

∆T is the constitutional supercooling degree;

$m_{L}$ is the liquidus slope;

$C_{0}$ is the concentration of Sr-modified element;

$k_{0}$ is the solute distribution coefficient;

$D_{L}$ is the solute diffusion coefficient;

x is the distance away from the solid-liquid interface; 

υ is the growth rate;

$G_{L}$ is the actual temperature gradient.

Generally, a higher constitutional supercooling degree ∆T leads to a more unstable solid–liquid interface, and easily results in the growth of cellular or even dendritic crystals. Increasing the concentration $C_{0}$ of the modifying element increases ∆T, which further facilitates bifurcation of the eutectic Si phase and leads to its refinement. As the eutectic reaction and the formation of compounds proceed, the crystallization-front (associated with Sr concentration in the Si phase) at the eutectic Si mass boundaries stops abruptly, which is accompanied by the reduction or disappearance of constitutional supercooling. As a result, Si crystal growth mode switches to the cellular crystal growth mode or plane unmodified growth mode, which confirms partial modification.

### 3.2. Influence of the Holding Time of Melt Modification Treatment on Modification Effect

**Figure 4:**
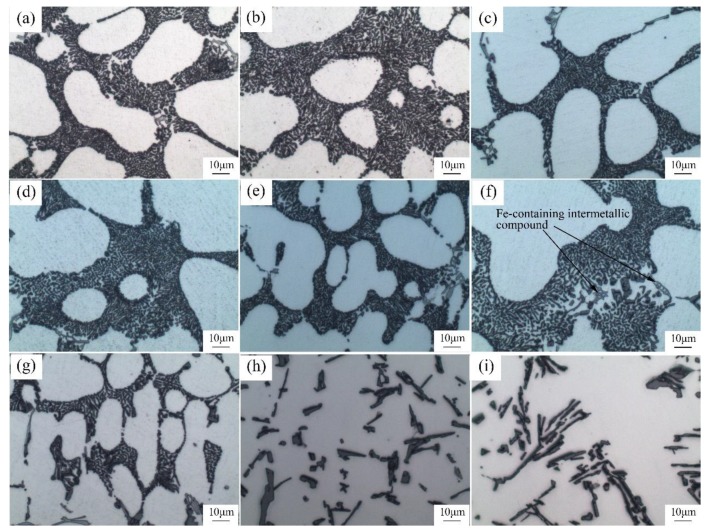
Morphology of the eutectic Si cast from the melt with 0.06 wt.%Sr addition holding for different times at 750 °C: (**a**) 10 min, (**b**) 20 min, (**c**) 30 min (**d**), 60 min, (**e**) 90 min, (**f**) 120 min, (**g**) 150 min, (**h**) 180 min, and (**i**) 240 min.

**Figure 5:**
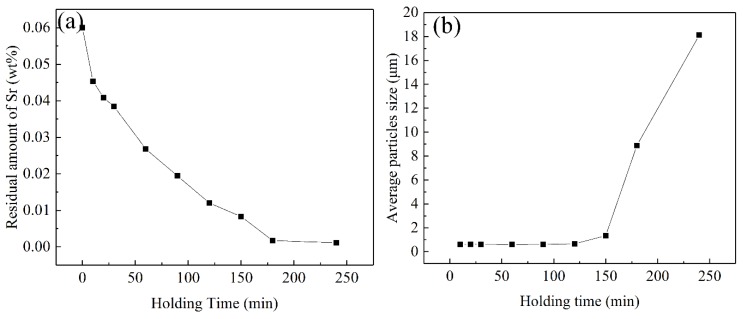
(**a**) The residual amount of Sr and (**b**) the average particles size of eutectic Silicon as function of the melt holding time.

According to Figure 4, in the A356 aluminum melt whose additive amount of Sr was 0.06%, the eutectic Si phase immediately achieved a satisfactory modification with a holding time of 10 minutes at 760 °C (Figure 4a). At longer holding times, the eutectic Si phase was further refined. At a holding time of 30 min, the refinement effect reached its peak. When the holding time of the melt was 120 minutes, the eutectic Si phase maintained its satisfactory modification effect. However, at a 150-minute holding time (see Figure 4g), the modification effect began to fade, and at a 180-minute holding time, the morphology of the eutectic Si phase became a bulky, massive one (Figure 4h). At a holding time of 240 minute, the morphology of the eutectic Si phase completely reverted to its unmodified state (Figure 4i). Obviously, Sr lost its modification potential, a phenomenon known as the fading effect [7,24], because of the amount of Sr which was lost. This is also confirmed by a composition test shown in Figure 5a. Figure 5a indicates that as the holding time of the melt increased, the actual residual amount of Sr in the melt gradually decreased. For example, with a 120-minute holding time, the residual amount of Sr was 0.012%; yet, the eutectic Si phase still demonstrated a satisfactory modification effect. At 150 minutes’ holding time, the modification effect began to fade and the residual amount of Sr was 0.0083%. At a holding times of 180 minutes, the residual amount of Sr decreased to 0.0017%, and the morphology of the Si phase deteriorated into plate-like bars. At 240 min, the morphology of the eutectic Si phase completely returned to its unmodified state. This was confirmed according to the average particles size of eutectic silicon, as shown in Figure 5b.

Thus, it can be inferred that the concentration of Sr in the melt had a critical value of ~0.012% (to have an effect on modification of the eutectic Si phase), which is very close to the value (equal to 0.01%) given by Fortini et al. [16]. Zarif et al. [15] also pointed out that only 50 ppm is good enough to alter the morphology of Si. The difference in the critical value from our result may be due to different alloys with different alloying elements and different smelting processes which lead to different loss levels of Sr. Therefore, to be on the safe side, 250–300 ppm [24,25] is usually preferred in typical foundry applications.

**Figure 6:**
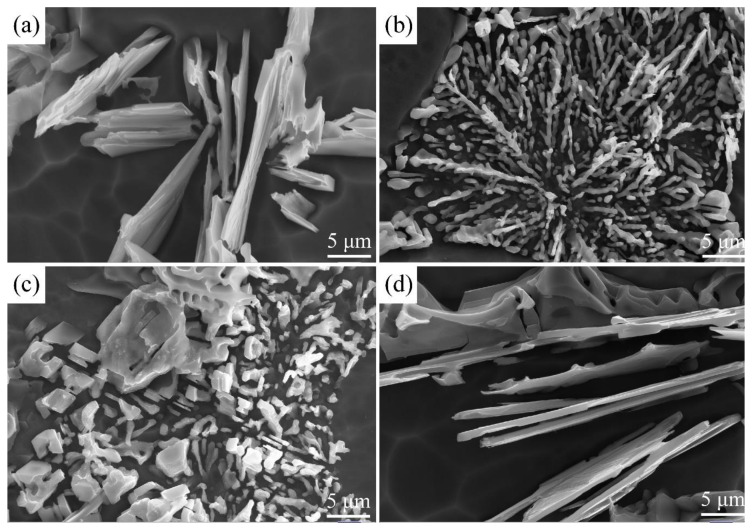
Stereoscopic morphology under SEM ((**a**) unmodified; (**b**) residual amount of Sr = 0.0384%; (**c**) residual amount of Sr = 0.0083%; (**d**) residual amount of Sr = 0.0011%).

Figure 6 shows the SEM images of the metal mold samples of an A356 aluminum alloy after deep corrosion by 15% NaOH solution. Figure 6a shows the stereoscopic morphology of the unmodified eutectic Si phase, which demonstrates a plate-like morphology. This observation agrees with the results of the 2D metallographic images (which demonstrated an acicular eutectic Si morphology) obtained using an optical microscope. Figure 6b shows numerous, well-modified, fine dendritic eutectic Si grains which formed when the residual amount of Sr was 0.0384% after a holding time of 150 min. It may be clearly seen that when the residual amount of Sr was >0.012%, the morphology of the eutectic Si phase changed from a plate-like morphology to a well-developed, bifurcated, dendritic morphology. Figure 6c shows the morphology of the Si phase obtained with a 150-minute holding time and a Sr residual amount of 0.0384%. Some dendritic particles of the eutectic Si phase are still seen, but most of them became large and coarse, with irregular morphologies. This suggested that as the holding time increased, the Sr modification effect became more negligible. Figure 6d shows that the morphology of the eutectic Si phase at a 240-minute holding time had a plate-like morphology (about 0.7 μm in thickness). These results again confirm that at holding times of over 240 minute, the eutectic Si phase experiences a complete failure in terms of modification, and reverts to its unmodified state.

### 3.3. Influence of Cooling Rate on Modification Effect

**Figure 7:**
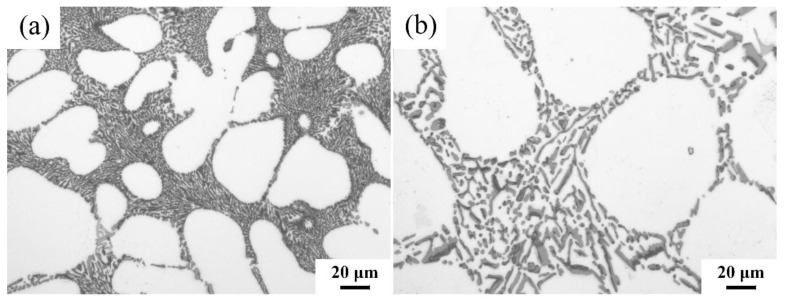
As cast microstructures of A356 alloys obtained by (**a**) metal and (**b**) sand molds.

Figure 7 shows the morphology of eutectic Si particles in hypoeutectic Al-Si alloys with the same Sr contents (0.06 wt % Sr), cast at different cooling rates and obtained using an iron mold and a sand mold, respectively. The eutectic Si phase in alloys cast using the iron molds with higher cooling rates showed satisfactory modifications. Meanwhile, in alloys cast using the sand mold with low cooling rates, the primary α (Al) phase became coarser and the eutectic Si phase demonstrated an incomplete modification, which is consistent with the conclusion of Sun et al. [26]. The residual amounts of Sr in iron mold castings and in sand mold castings were 0.026 ± 0.002 and 0.018 ± 0.002, respectively. 

The mechanism of cooling rates on the eutectic Si phase modified with Sr is still not very clear. Sun et al. [26] thinks that a low cooling rate decreased the release of Sr in the A356 alloy, in which there are not enough free Sr atoms and the diffusion distance between the Sr atoms and the Si surface would be increased, therefore, deteriorating the metamorphic effect of Sr. But Lan et al. [20] reported that the cooling rate did not influence the distribution of Sr in Al-Si alloys. This was confirmed by “superimposed” processing for a constitutional distribution map acquired using electron probe micro-analysis (EPMA) and obtaining quantitative data on the Sr distribution on the eutectic Si phase surface and α-Al surface for the corresponding alloys prepared at different cooling rates. 

What is clear is that the Sr modification for eutectic Si is sensitivity to cooling rate. It can be inferred that Sr-modified eutectic Si has a critical cooling rate. As a result, to obtain a satisfactory modification of eutectic Si for heavy wall or sand mold castings, one needs to consume more Sr than for iron mold castings.

## 4. Conclusions

The relationship between the Sr residual amount and the morphology of the eutectic Si phase in hypoeutectic Al-Si alloy is discussed. A general conclusion can be drawn that there is a critical value of ~0.01 wt % Sr in the melt to have a good effect on the eutectic Si phase modification. So, eutectic silicon can be modified well with the smallest Sr addition by optimizing the smelting process and reducing the burning loss of Sr, in industrial practice. This has good economic benefits and technical feasibility.

(1) The effect of a complete modification of the eutectic Si phases in A356 is noticeable with a Sr additive amount equal to 0.03 wt %. A higher amount of Sr added to the melts (~0.04–0.06 wt %) did not achieve more significant improvements;

(2) When 0.06 wt % of Sr was added to the Al-Si alloy melts, a satisfactory modification effect could be obtained by holding the samples at 750 °C for 10–120 min. However, at holding times of ~150 min, the modification effect started to fade, and at 240 min, the modification effect disappeared completely;

(3) The modification effect of the eutectic Si phase formed in A356 aluminum melt was dependent on the residual amount of Sr. At least 0.01 wt % of the residual amount of Sr is needed to obtain good morphology of eutectic Si in an A356 cast in an iron mold;

(4) The modifying abilities of Sr were remarkably affected by the cooling rate. There was an incomplete modification of the eutectic Si phase in an A356 alloy with the addition of 0.06 wt % Sr cast in a sand mold, even though the residual amount of Sr was 0.018 ± 0.002 wt %, i.e., more than that of the critical residual amount 0.01 wt % Sr for an A356 alloy cast in an iron mold.

## Figures and Tables

**Table 1:** Chemical composition of A356 aluminum alloy (wt.%).

Alloy	Si	Mg	Fe	V	Al
A356	7.580	0.341	0.101	0.032	Balance
